# Narratives of experience of mental health and illness on healthtalk.org

**DOI:** 10.1192/pb.bp.115.052217

**Published:** 2016-10

**Authors:** Jo Kidd, Sue Ziebland

**Affiliations:** 1 healthtalk.org, Oxford, UK; 2 University of Oxford, UK

## Abstract

Online health information is increasingly popular and may bring both benefits and potential harm to users with mental health problems. The encouragement of harmful behaviour among this population is a particular concern. The website healthtalk.org provides the benefits of shared experience by publishing excerpts from rigorous research interviews with patients, contextualised with medical information. This article sets out evidence for the positive and negative effects of online mental health information and describes the methodology behind healthtalk.org, with an overview of the mental health information it provides and how it can benefit patients and health professionals.

The internet has revolutionised how people deal with symptoms and illness. Almost half of UK adults now search for health information online, an increase from 18% in 2007.^1^ Mental health resources have proliferated on the web since the mid-1990s. Patients, their friends and families can find detailed information on a range of common, and less common, mental health conditions from myriad reliable (and less reliable) sources.^2^ As well as this more ‘traditional’ health information, patients can now access online cognitive-behavioural therapy (CBT) interventions, connect with other patients in online support groups and fora, and read or view personal accounts and blogs. The web can also provide a tool for people with mental health conditions to become activists, raising awareness and challenging stigma around mental illness.^2^

Although using the internet for health information and support is now routine, normal practice, concerns remain about the potential for harm, especially for people who are considered vulnerable. Pro-anorexia (or ‘pro-ana’) websites may cause harm by providing ‘tips’ and ‘thinspirational’ imagery which encourage harmful behaviour and exacerbate symptoms.^3^ Evidence is equivocal about the influence of chat rooms for people who are self-harming or considering suicide: on the one hand, these fora may support people who are socially isolated and help them cope, but some researchers have concluded that such chat rooms may do harm by ‘normalising’ self-harming behaviour or suicidal intent.^4^ In 2012, micro-blogging platform and social network Tumblr was implicated in the suicide of British teenager Tallulah Wilson. Tallulah had been blogging about her self-harm and interacting with others who self-harmed. Following the case, Tumblr changed their procedures to encourage users to report content that encouraged self-harm and sharing of self-harm images. The internet clearly presents both risks and opportunities for prevention of harm in young adults.^5^

The reliability of mental health information found online has also raised concerns. A study of mental health websites popular in the USA concluded that more than two-thirds of information provided on common mental health conditions could be considered as ‘good’ or ‘better quality’ and that this has improved over time.^6^

One of the most transformative effects of the internet revolution is that people can now access, at any time of day or night, accounts of how others have dealt with the same condition. They can do this in private, without anyone knowing that they are doing so. They can learn through the experiences of others how to manage their own condition, decide when and whom to consult and how to deal with health and care services, as well as sharing and gaining peer support.^7^ This may be especially pertinent to those with a mental health condition, for whom stigma can be a barrier to seeking help.^8^ Peer support as an intervention has been shown to help reduce symptoms of depression^9^ and peer support delivered online was associated with improved experience and emotions in the case of people with mental health conditions and long-term health conditions.^10^ However, more research is needed to understand the efficacy of online peer support for mental health conditions.^11^

## About healthtalk.org

Based on a belief in the value of peer support, as well as the recognition of the value of a broad sample of experiences and a ‘curated’ delivery, healthtalk.org publishes analyses and extracts from interview studies using narrative methods. Most of the interviews are video recorded, if the participant's consent has been given. The website has some of the features of online forums (a broad range of experiences, peer support, the issues that matter to patients) but as it is based on carefully conducted research with broad samples of participants, it provides a balanced, evidence-based reflection of what is important to patients, presented through video and audio interview clips as well as written material.^12^

The website healthtalk.org is the product of a unique collaboration between DIPEx, a registered UK charity, and the Health Experiences Research Group (HERG) at the University of Oxford's Nuffield Department of Primary Care Health Sciences. The two have worked together for 15 years to offer support and information on a range of different health issues from the perspective of those who experience them first-hand.^13^

The website was the brainchild of Oxford general practitioner Dr Ann McPherson CBE and Dr Andrew Herxheimer, clinical pharmacologist, founding editor of the *Drugs and Therapeutics Bulletin* and emeritus fellow of the Cochrane Collaboration. They were inspired to collect a database of patient stories by their own health experiences in the late 1990s. Dr McPherson had been diagnosed with breast cancer and Dr Herxheimer was scheduled for knee replacement surgery. Both were aware of the value of hearing the real stories of other patients, as well as learning from the results of randomised controlled trials.

Originally intended to be a collection of individual written accounts, it was soon realised that the project would be much more useful if it represented a wide range of experiences of each condition covered and would be more inclusive and accessible if based on digitally recorded interviews rather than written accounts. Information on each health condition included in the project is based on a national, diverse sample of interviews collected and analysed by skilled and trained qualitative researchers. The researchers travel all over the country and interview people in their own homes, using open-ended ‘narrative’ methods that encourage the participant to talk about what matters to them (rather than what matters to researchers and clinicians).

A website team that sits within the charity DIPEx works with the researchers to publish video and audio extracts and analysis from the interview collections intended for patients, public, family members and professionals. The first sections, on experiences of prostate cancer and experiences of high blood pressure, were published on the former DIPEx website in 2001. Fifteen years later the site is called healthtalk.org, includes nearly 100 different conditions (including several mental health collections) (Box 1) and has sister projects in 12 countries (dipexinternational.org).

### Mental health topics on healthtalk.org

The website covers a number of mental health issues: depression, depression and low mood in young people, eating disorders in young people, psychosis, antidepressants, mental health experiences of ethnic minorities and ethnic minority carers, parents' experiences of self-harm and, the most recent addition, patients' experiences of electro-convulsive therapy (ECT) (links are included in Box 1). For each of these issues, there are video, audio and written excerpts from interviews with around 40 people who have experienced the issue personally or through a family member. The research team analyse the interviews and write thematic summaries on topics such as diagnosis, work, support, making decisions about treatment, and impact on friends and family. Each of these themes is illustrated with different experiences and perspectives from the interviews so that people who use the site can find experiences that resonate with their own (albeit perhaps not voiced by someone whose experiences they might have assumed would be like their own).

This broad range of experience was particularly important in the case of the antidepressants section, where interviewees talked about how people react differently to depression medicines. Stuart (52 years old) had tried a number of different antidepressants over the years:

‘From my experiences they work very differently for different people and you can't tell until you've started taking the drugs what they're going to do for you, what the therapeutic effect is going to be, you can't say what side effects you're going to get, if any’.

John, 84, was diagnosed with depression in his 30s, and prescribed diazepam. A few years before being interviewed, he was prescribed fluoxetine for the first time: ‘The black clouds lifted’ he said. In contrast, Peter (aged 34) said that taking venlafaxine after being on fluoxetine without success was ‘like waving a magic wand’. Lucy (aged 21) came off fluoxetine after 6 months because she felt dizzy and ‘distant’ from her emotions. She told the interviewer that she realised only later that different drugs were available and advised that patients should talk to their doctors if they are not getting on with their prescription or if they would like to explore other treatment options.

**Box 1** Mental health collections on healthtalk.org

- Antidepressants (www.healthtalk.org/peoples-experiences/mental-health/experiences-antidepressants/topics)
- Depression (www.healthtalk.org/peoples-experiences/mental-health/depression/topics)
- Depression (in Australia) (www.healthtalk.org/peoples-experiences/mental-health/experiences-depression-and-recovery-australia/topics)
- Eating disorders in young people (www.healthtalk.org/young-peoples-experiences/eating-disorders/topics)
- Electroconvulsive treatment (www.healthtalk.org/peoples-experiences/mental-health/electroconvulsive-treatment/topics)
- Low mood and depression in young people (www.healthtalk.org/young-peoples-experiences/depression-and-low-mood/topics)
- Mental health: ethnic minority experiences (www.healthtalk.org/peoples-experiences/mental-health/mental-health-ethnic-minority-experiences/topics)
- Mental health: ethnic minority carers' experiences (www.healthtalk.org/peoples-experiences/mental-health/mental-health-ethnic-minority-carers-experiences/topics)
- Experiences of psychosis (www.healthtalk.org/peoples-experiences/mental-health/experiences-psychosis/topics)
- Self-harm: parents' experiences (www.healthtalk.org/peoples-experiences/mental-health/self-harm-parents-experiences/topics)

## How healthtalk.org is used

‘I'm 20 years old and have been struggling with depression since I can remember, and have struggled even more with trying to get those around me to understand how I feel. I would try to explain it to them and they still wouldn't get it, making me feel more alone. I sent my parents the link to this page and had them watch the videos, and now they (and I) know that I am not alone with my illness and the way it makes me feel. Now instead of shaming me for the way my depression makes me live, they understand that it can be crippling and have decided to help me. Thank you so much, this information saved my life.’

This message was received by the healthtalk.org team in 2015. It came from Alice, a young woman who had been looking at a page entitled ‘What does depression feel like: social & physical experiences’. Her comment demonstrates the value of being able to access online personal stories of other ordinary young people who, like Alice, are experiencing depression.

Approximately 5 million people visited healthtalk.org in 2015, of whom half a million were seeking mental health information. A visitor survey suggests that the patient stories have a real impact too: 8 in 10 people agreed or strongly agreed that they felt better informed after viewing the information and 7 out of 10 agreed or strongly agreed that they felt better prepared for, and less alone in, what they were facing. Furthermore, 85% of people reported that they agreed or strongly agreed that the information on healthtalk.org is better than they have seen elsewhere and 8 out of 10 agreed or strongly agreed that healthtalk.org provides information on issues that they had not found covered elsewhere. Examples include emotional lability after stroke, eating disorders in young men and the parents' perspective on self-harm in young people. Arguably, the use of qualitative, narrative research methods also enables the team to tackle issues with a depth that might not be available elsewhere. Finding out from patients what was important to them allows the research team to tap into the issues that might help other patients.

## How healthtalk.org can help health professionals

Although the vast majority of people who visit the website are seeking information for themselves or a loved one, about 5% of the audience use the website for work. Mental health professionals can find out what patients in general think of the care they have received, what works and what does not. The following quotes are from the website's section on depression in Australia and illustrate different perspectives on the care that patients received. We do not reveal the names of health professionals or hospitals on the website and are careful to remove such identifiers from the interview extracts before they are published on the website.

‘We have a mutual respect of each other. [My psychiatrist] is quite a remarkable person. The first dog I had, my psychiatrist got me to have because I was so unwell I shouldn't live alone. He was so scared I was going to kill myself and he thought that if I had a dog with me, I might think again before killing myself and having the dog locked in the house with a dead person … I just couldn't bring myself ever to do it when the dog was there. So, so it was very smart thinking from my doctor and it worked a real treat, yeah.’ (Suzie).

‘When I cried in [the psychiatrist's] office she just sort of looked at me as if I was a freak. When she said, “You've got postnatal depression” and I absolutely burst into these tears … I think she actually said … “Well, what did you think I was going to say?” And I thought that wasn't particularly supportive’ (Emma).

The website is used across the globe in the teaching and training of health professionals and professionals in other disciplines including the police and social care. We are aware that it is used in teaching in almost all of the medical schools in the UK. Recently a function called ‘Scrapbooks’ was added to the website that allows teachers and learners to create their own collections of video clips and articles from across healthtalk.org. Videos added to scrapbooks will appear in a playlist. The feature has proved popular – some teachers have set the students the task of creating their own scrapbooks.^14^

Not only are the healthtalk.org resources used in training professionals but there is evidence to show that these national collections of patients' experiences can be used as part of studies to improve how health services are delivered. A recent study used an ‘accelerated’ version of a co-design approach called ‘experience-based co-design’. They found that using ‘trigger films’ based on the pre-existing interviews collected by HERG in national collections was as effective for stimulating local service improvement initiatives as previous studies that had included a lengthy process of local data collection with patients and staff. They found that using the HERG collections for the trigger films did not adversely affect engagement with National Health Service (NHS) staff and could make the process less threatening; the films also worked well as trigger for discussion and led to similar improvement activities to those implemented in earlier studies after using local narratives.^15^ The team behind this paper have created a trigger film based on experiences of psychosis and another one based on young people's experiences of depression is planned.

The interviews collected for the study of experiences of psychosis were also used to inform the National Institute for Health and Care Excellence (NICE) clinical guidelines on service user experience for people using adult NHS mental health services, published in 2012.^16^

## Conclusion

healthtalk.org sits alongside other respected UK providers of online mental health information such as Mind (www.mind.org.uk), the Royal College of Psychiatrists (www.rcpsych.ac.uk), Rethink (www.rethink.org), CALM (www.thecalmzone.net) and the Mental Health Foundation (www.mentalhealth.org.uk) in serving a population of people who live with mental health conditions and the general public, who increasingly turn to the internet for information about health.^1^ The work of these organisations helps individuals to seek support and practical information on mental health issues, in private and at a time convenient to them.^8^

In summary, healthtalk.org complements existing resources to offer in-depth information on people's experiences of mental illness, through the rigorous qualitative research methods of the University of Oxford's Health Experiences Research Group at the Nuffield Department for Primary Care Health Sciences.

Future areas for research that will be made available through healthtalk.org include a project on the depression experiences of young adult Americans, which is in progress by colleagues at the University of Wisconsin, Madison. They, and other members of the DIPEx International collaboration, are keen to conduct studies on many other health conditions, including mental illness, and are currently seeking funding for projects on bipolar affective disorders, obsessive-compulsive disorder and post-traumatic stress disorder.
